# Endurance Training in Olympic Winter Sports: A Narrative Review of the Current Literature and Future Research Priorities

**DOI:** 10.1111/sms.70220

**Published:** 2026-02-11

**Authors:** Billy Sperlich, Hans‐Christer Holmberg

**Affiliations:** ^1^ Integrative and Experimental Exercise Science & Training, Institute of Sport Science University of Würzburg Würzburg Germany; ^2^ Division of Machine Elements Luleå University of Technology Luleå Sweden; ^3^ Department of Physiology and Pharmacology, Biomedicum C5 Karolinska Institutet Stockholm Sweden; ^4^ School of Kinesiology University of British Columbia Vancouver Canada

**Keywords:** adaptive phenotyping, duarability, fatgability, fatigue, load‐response modeling, recovery, training responsiveness

## Abstract

The Milano‐Cortina 2026 Winter Olympics present an opportunity to synthesize evolving paradigms in endurance training within the broader context of long‐term athlete development. As Olympic winter sports span from endurance‐limited events to disciplines in which aerobic fitness primarily serves as a feeder capacity, a single training model is insufficient. In this narrative review, we propose a dual framework: (1) a demand‐driven, athlete‐centered, data‐supported model for Endurance‐Limited sports (e.g., cross‐country skiing, biathlon) and (2) a Feeder‐Function model for sports in which endurance primarily supports recovery, training tolerance, and resilience (e.g., freestyle skiing, snowboarding, sliding sports). Within this framework, we narratively synthesize and critically evaluate the literature across key domains, including individualized volume–intensity architectures, the integration of concurrent strength training, and the strategic use of multimodal stress stacking (e.g., hypoxia, heat). We further address the operationalization of emerging performance constructs such as durability, fatigability, resilience, and repeatability. We also present a heuristic tier framework describing when endurance acts as a primary performance limiter versus a supporting capacity across Olympic winter sports. Subsequently, we examine the role of advanced technologies, from multisensor wearables and analytics to mechanistic approaches (e.g., multiomics), highlighting their potential to shift practice from passive monitoring to active, individualized modeling. Future research priorities include validating field‐based operational metrics, defining minimal effective endurance doses for feeder‐function sports, and developing interpretable, athlete‐centered decision‐support tools. By aligning sport‐specific demands with individualized, evidence‐informed prescription, this dual‐framework approach offers a perspective to guide interpretation and future applied work for scientists, coaches, and athletes preparing for Milano‐Cortina 2026 and beyond.

## 1 Introduction

Seventy years after first hosting the Winter Olympics in 1956, Cortina d'Ampezzo will cohost the Milano‐Cortina 2026 Games. This return offers a vantage point to assess the evolution of elite performance in Olympic winter sports. As outlined in Table 1, the 2026 program is markedly broader than its 1956 predecessor, featuring a ~5‐fold increase in medal events and achieving gender parity across nearly the full spectrum of disciplines.

**TABLE 1:** Evolution of Olympic winter sports disciplines: A comparison of the men's and women's programs between the 1956 Cortina d'Ampezzo Games and the upcoming 2026 Milano‐Cortina Games.

Sport	Men's disciplines 1956	Men's disciplines 2026	Women's disciplines 1956	Women's disciplines 2026
Alpine skiing	Downhill, Giant slalom, Slalom	Downhill, Super‐G, Giant slalom, Slalom, Team combined	Downhill, Giant slalom, Slalom	Same as men
Biathlon	—	Sprint, Pursuit, Individual, Mass start, Relay, Mixed relay	—	Same as men
Bobsleigh	2‐man, 4‐man	2‐man, 4‐man	—	Monobob, 2‐woman
Cross‐country skiing	15 km, 30 km, 50 km, 4 × 10 km relay	10 km, 20 km, 50 km, Sprint, Team sprint, 4 × 7.5 km relay	10 km, 3 × 5 km relay	Same as men (full parity)
Curling	—	Men's team, Mixed doubles	—	Women's team, Mixed doubles
Figure skating	Singles, Pairs (mixed)	Singles, Pairs (mixed), Ice dance, Team event	Singles, Pairs (mixed)	Same as men
Freestyle skiing	—	Moguls, Dual moguls, Aerials, Halfpipe, Slopestyle, Ski cross	—	Same as men
Ice hockey	Yes	Yes	—	Yes
Luge	—	Singles, Doubles, Team relay	—	Singles, Doubles, Team relay
Nordic combined	Yes	Individual normal hill, Individual large hill, Team sprint	—	—
Short‐track speed skating	—	500 m, 1000 m, 1500 m, Relays	—	Same as men
Skeleton	—	Individual	—	Individual
Ski jumping	Medium relay#	Normal hill, Large hill, Super team, Mixed team		Normal hill, Large hill, Mixed team
Ski mountaineering	—	Sprint, Mixed relay	—	Sprint, Mixed relay
Snowboarding	—	Halfpipe, Slopestyle, Big air, Parallel GS, Snowboard cross	—	Same as men
Long‐track speed skating	500 m, 1500 m, 5000 m, 10 000 m	Same + Mass start, Team pursuit	—	500 m, 1000 m, 1500 m, 3000 m, 5000 m, Mass start, Team pursuit
Total medal events	18 events	~58 events	6 events	~58 events

*Note:* The 2026 program illustrates the introduction of “sprint” formats, mixed‐gender relays, and new disciplines (e.g., Ski mountaineering). #: Designated based on current FIS hill size standards (K‐point approx. 72m).

Crucially, this expansion has diversified the physiological demands of preparation and competition. Modern winter Olympic sports have extended beyond traditional steady‐state endurance to include high‐intensity sprint and multiround formats (e.g., cross‐country skiing, ski mountaineering) and intermittent, technically complex disciplines (e.g., mogul skiing and snowboard cross). In parallel with broader scientific and technological advancements, elite preparation has increasingly shifted, particularly over the last two to three decades, from generalized, experience‐driven approaches toward more data‐informed and highly specialized training models. Importantly, although maximal aerobic power (V̇O_2_max) in elite winter endurance athletes has remained relatively stable over the past five to six decades [1, 2, 3, 4], performance improvements appear to be driven less by further increases in V̇O_2_max and more by its effective utilization, including the efficiency with which metabolic energy is converted into mechanical power and sustained under sport‐specific constraints (e.g., technique‐ and terrain‐specific demands, repeated high‐intensity efforts, and fine motor control under fatigue). However, the role of endurance within this heterogeneous competitive landscape remains variably interpreted, particularly outside endurance‐dominant sports.

Over the past decades, a central “game changer” in endurance winter sports has been the increasing specificity of endurance training [5, 6]. This shift is reflected in the greater use of sport‐specific modalities (e.g., roller skiing in cross‐country skiing and biathlon) and the targeted development of sport‐specific skills under physiological load (e.g., simulated competition demands, shooting performance in biathlon), rather than a primary focus on increasing maximal aerobic power per se. These developments have been facilitated by advances in snow and ice preparation, equipment, training environments, and measurement technologies (e.g., heart rate and lactate monitoring, video analysis, GPS and IMUs), enabling more precise characterization and quantification of sport‐specific metabolic, mechanical, and technical work demands. To address this complexity and operationalize the varying roles of aerobic fitness, we developed a heuristic tier framework that categorizes Olympic winter disciplines into five tiers based on their predominant role of endurance for competitive outcome (Table 2).

**TABLE 2:** Conceptual framework for the role of endurance across Olympic Winter Sports in Milano Cortina 2026.

Tier	Role of endurance	Winter Olympic events	Typical duration	Intensity metrics	Endurance training priority & focus	Exercise modes for endurance training
Tier 1—Maximal aerobic endurance (prolonged)	Maximal aerobic power is a major performance determinant; high aerobic demand is sustained during prolonged and/or repeated efforts (even‐ or variable‐paced).	XC skiing; Biathlon; LTS (10 000 m; men)	~3–130+ min (event‐dependent; shortest formats involve repeated high‐intensity work^a^)	Mean HR ~90% HRmax with frequent peaks > 95% HRmax; V̇O_2_ typically ~80%–95% V̇O_2_max (terrain/format dependent)	“Train endurance to compete.”	Specific (XC/Biathlon: skiing on snow; LTS: skating on ice); Semispecific (XC/Biathlon: roller‐skiing, double‐poling ergometer; LTS: inline skating, slide board); Nonspecific (running, cycling, Nordic walking/hiking). Relative emphasis differs by discipline (XC/Biathlon predominantly specific/semispecific; LTS endurance volume is predominantly off‐ice).
Tier 2—Endurance dominant (severe intensity)	Endurance‐dominant at high‐to‐severe intensities; sustaining high aerobic power depends on pacing control and fatigue resistance (pace variability is event‐dependent)	LTS (1500–5000 m); Nordic combined (XC segment); SkiMo (sprint format)	~2–30 min (race/segment; event‐dependent); SkiMo sprint involves repeated heats, with higher cumulative competition exposure for finalists.	Mean HR typically ~88%–95% HRmax (more even‐paced in time trials; more variable in tactical/terrain‐driven events); V̇O_2_ ~75%–95% V̇O_2_max (near‐maximal in key phases)	“Train endurance to sustain severe‐intensity output and ensure fatigue resistance (including responsiveness to pace changes when required).”	Specific (NC/SkiMo: skiing on snow; LTS: skating on ice); Semispecific (NC/SkiMo: roller‐skiing, uphill hiking/running with poles; LTS: inline skating, slide board); Non‐specific (running, cycling, Nordic walking). Relative emphasis mirrors Tier 1 (NC/SkiMo predominantly specific/semispecific; LTS endurance volume predominantly off‐ice).
Tier 3— High‐intensity mixed demands (round‐based/intermittent or continuous)	Supports repeated high‐intensity work and fatigue resistance; preserves technical/decision‐making quality, enables rapid recovery between high‐power bouts and facilitates maintenance of output across sustained mixed‐demand efforts.	LTS (1000 m); STS (1000–1500 m); Alpine skiing (GS, SG, DH); Freestyle skiing (ski cross, moguls, slopestyle); Snowboard (PGS, snowboard cross, slopestyle); Ice hockey; Figure skating	Single race/run/program: ~0.5–5 min (event‐dependent); repeated runs/bouts: ~20–180 s; total competition exposure in round−/shift‐based formats: ~60–150+ min (incl. stoppages; format‐dependent).	High physiological strain: HR typically reaches ~85%–95% HRmax during/near the end of efforts (often persisting into recovery), with repeated peaks > 95% HRmax in round−/shift‐based disciplines; substantial oxygen deficit/glycolytic contribution per bout, with cumulative metabolic strain across rounds/shifts and successive runs (sport dependent)^b^.	“Train endurance to be able to train at high intensity—so you can compete successfully.”	Specific (sport‐specific on snow/ice as feasible); Semispecific (skating‐based modalities, slide board, sport‐relevant ergometers); Non specific (cycling, rowing, running, hiking). Primary emphasis is commonly semispecific to non‐specific, with specific exposure constrained by access (snow/ice availability), travel/logistics, and injury/impact risk.
Tier 4—Sprint/explosive dominant (short)	Performance constrained by sprint/anaerobic power and technical execution; endurance supports recovery between heats/runs and enables higher‐quality technical repetitions in training.	LTS/STS (500 m); Alpine skiing (SL); Freestyle skiing (halfpipe); Snowboard (halfpipe)	~20–70 s per run/race (discipline‐dependent; often repeated heats/rounds or 2‐run formats).	Very high anaerobic strain: HR typically reaches ~90%–100% HRmax by late effort or early recovery; blood lactate accumulation is often high (discipline dependent).	“Train endurance to tolerate volume and recover between explosive runs.”	Specific (sport‐specific on snow/ice as feasible; alpine athletes commonly train across disciplines); Semispecific (skating/inline skating, slide board, ergometer‐based conditioning); Non—specific (cycling, running, rowing, hiking/stair climbing).
Tier 5—Skill/strategy‐dominant (endurance supportive)	Performance is skill/strategy‐ and power‐limited; endurance supports robustness, recovery, and training‐load tolerance.	Freestyle skiing (aerials, big air); Snowboard (big air); Curling; Sliding sports (bobsleigh, skeleton, luge); Ski jumping	Decisive effort < 10 s (start/take‐off or delivery; per attempt/shot); competition exposure ~30–180 min (discipline−/format‐dependent).	HR often elevated (arousal/isometric load) despite low aerobic contribution to decisive efforts; aerobic demand mainly reflects total session exposure.	“Train endurance for general fitness and robustness—supporting training tolerance—not as a primary performance limiter.”	Nonspecific (running, cycling, rowing; low‐impact cross‐training as needed).

*Note:* Winter Olympic disciplines are classified into five tiers according to the *predominant* role of endurance for competitive outcome—ranging from endurance as a primary physiological performance limiter (Tiers 1–2; most pronounced in Tier 1) to a supportive capacity for recovery and training‐load tolerance (Tier 5). The framework is heuristic and integrates available sport‐specific physiological evidence with sport demands analysis and author expertise. Where direct sport‐specific evidence is limited, classifications were informed by targeted expert consultations. Specific disciplines listed are representative examples. Reported values represent indicative ranges and qualitative patterns (not universal prescriptions) and may vary with terrain, tactics, competition format, individual athlete profiles, and event specialization. Disciplines may reasonably span adjacent tiers, particularly in round−/heat‐based formats and events with stochastic pacing.

Abbreviations: DH, downhill; GS, giant slalom; h, hr (s); HR, heart rate; HRmax, maximal heart rate; LTS, long‐track speed skating; min, minute(s); NC, Nordic combined; PGS, parallel giant slalom; s, second(s); SG, super‐G; SkiMo, ski mountaineering; SL, slalom; STS, short‐track speed skating; V̇O_2_, oxygen uptake; V̇O_2_max, maximal oxygen uptake; XC, cross‐country skiing.

^a^ XC sprint skiing finalists complete four rounds (qualification + three heats); ~3 min per effort (course dependent); total exposure ~1.5–2.5 h (including recovery).

^b^ HR can overestimate metabolic load in technically constrained/isometric disciplines (Tiers 3–5); where relevant, HR should be interpreted alongside pacing, external workload, and lactate/oxygen‐uptake patterns.

**FIGURE 1:**
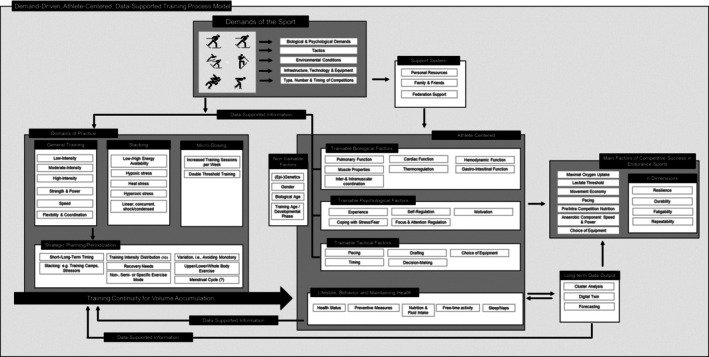
Illustration of factors involved in a demand‐driven, athlete‐centered, data‐supported training process of sports primarily determined by endurance capacity. The framework demonstrates how sport‐specific demands and individualized athlete characteristics are integrated into strategic planning, with a continuous data‐feedback loop refining the key determinants of competitive success (e.g., resilience, durability).

Tiers 1–2 (Endurance‐Limited) encompass disciplines such as cross‐country skiing and biathlon, where aerobic power, movement efficiency, and durability are primary physiological determinants of performance. These disciplines require a demand‐driven, athlete‐centered model in which training is individualized to the athlete's physiological profile to maximize their adaptive potential (Figure 1).

For these endurance‐limited disciplines, the past decade has yielded a wealth of knowledge, informed both by long‐standing research in, for example, cross‐country skiing and by transfer from extensively studied summer sports such as cycling and running. Contemporary evidence has refined our understanding of training intensity distribution (e.g., polarized vs. pyramidal models), altitude and hypoxic adaptation strategies, and the molecular regulation of endurance adaptation. At the same time, this body of work increasingly emphasizes that performance gains are more closely linked to improvements in efficiency, durability, repeatability, and fatigue resistance than to further increases in maximal aerobic power per se. A key objective of this review is to synthesize these modern developments and contextualize them within the distinct constraints of winter sports.

**FIGURE 2:**
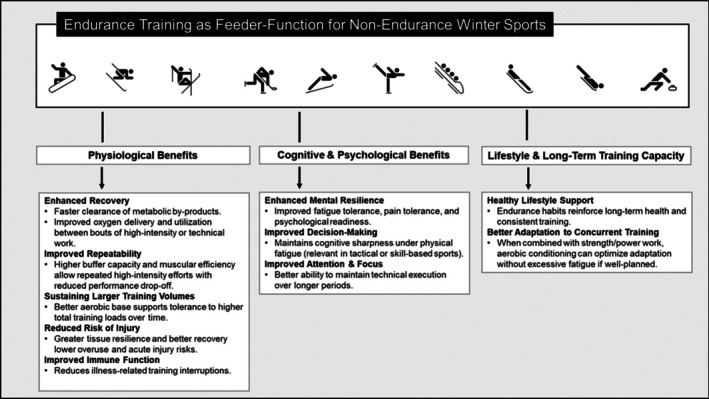
Endurance training as feeder‐function of Olympic winter sports not primarily limited by endurance capacity.

In contrast, Tiers 3–5 (Endurance as Feeder‐Function) encompass disciplines in which endurance is not a primary performance limiter, ranging from alpine skiing to sliding sports and curling, but instead functions, to varying degrees, as supporting capacity. In these disciplines, endurance training is strategically employed to enhance training tolerance (“the ability to train,”) specifically to facilitate recovery, support repeated high‐intensity efforts and technical work, and maintain performance robustness across the competitive season (Figure 2).

This spectrum from endurance‐limited to feeder‐function sports has fundamental implications for how training is structured, how adaptations are prioritized across the season, as well as for how evidence from endurance research is translated into sport‐specific practice. Over the past decade, advances in wearable technology [7], real‐time data analytics [8, 9], and cellular exercise physiology [10] have accelerated a shift from anecdote‐driven approaches toward systematic, data‐supported models, allowing more precise alignment between sport‐specific demands and athlete‐specific needs. These tools have been particularly influential in enabling more specific endurance training by quantifying terrain‐, speed‐, technique‐, and fatigue‐dependent work demands, thereby supporting a more precise alignment between sport‐specific requirements and athlete‐specific needs.

Consequently, this narrative review proposes two complementary conceptual frameworks: (i) a “Demand‐Driven, Athlete‐Centered, Data‐Supported Model” for endurance‐limited sports (Tiers 1–2); and (ii) a “Feeder Function‐Oriented Model” (Figure 2) for sports in which endurance is not the primary limiter (Tiers 3–5).

Accordingly, our aims here are twofold: (i) to synthesize current research on the physiological determinants and training strategies underpinning both the endurance‐limited (Tiers 1–2) and feeder‐function (Tiers 3–5) models; and (ii) to outline priority areas for research and innovation that will inform preparation strategies leading into and beyond the Milano‐Cortina 2026 Olympic cycle.

## 2 Method

This narrative review was prepared as part of a multi‐paper special issue addressing scientific and applied topics relevant to the preparation for the Olympic Winter Games Milano‐Cortina. The review synthesizes current knowledge on endurance training across Winter Olympic sports, including endurance‐limited disciplines and sports in which endurance primarily serves a feeder function.

We conducted structured, topic‐driven searches in PubMed, Web of Science, and Google Scholar between April and October 2025 using combinations of keywords related to elite endurance training and Olympic preparation in winter sports. Search terms covered environmental stressors (e.g., altitude and heat), concurrent training (e.g., strength, endurance), recovery, durability, resilience, monitoring/technology, and athlete health, and were refined iteratively as the manuscript evolved to capture emerging concepts relevant to the proposed conceptual frameworks. Reference lists of key reviews, consensus statements, and position papers were manually screened to identify additional sources. We prioritized peer‐reviewed publications in English from 2005 to 2025, while including relevant seminal older works when necessary for mechanistic or historical context. Study selection emphasized performance relevance focusing on athletes classified as “highly trained/national level” to “world class” [11] (where classification frameworks were available). We excluded studies on untrained, pediatric, or clinical populations lacking direct elite‐performance relevance.

Identified literature was mapped to three domains: (1) training methodologies, (2) recovery/health, and (3) technology/monitoring. Applied evidence (e.g., case studies, federation and coaching reports) was selectively integrated to provide ecological validity where controlled trials were lacking. Consistent with a narrative approach, we did not perform a systematic search or meta‐analysis. Instead, synthesis emphasized conceptual coherence and applicability to Olympic preparation. Limitations of this strategy include potential language bias and the non‐exhaustive nature of the review. Furthermore, we recognize a significant disparity in the evidence base; robust data exists for endurance‐limited sports (e.g., cross‐country skiing), while feeder/support‐function sports (e.g., snowboarding) rely more heavily on extrapolated physiological principles. These gaps are highlighted throughout the text as priority areas for future research. The tier classification in Table 2 was developed as a heuristic framework through iterative author synthesis integrating sport‐specific physiological literature with sport demands analysis and author expertise; where direct sport‐specific evidence was limited, classifications were informed by targeted expert consultations with elite coaches and high‐performance staff in selected sports.

## 3 Sport‐Specific Demands: Defining the Dual‐Framework Model

In the context of Olympic winter sports, endurance denotes the integrated capacity to sustain power output, resist fatigue, and recover rapidly between bouts. These sports span a broad spectrum, ranging from the prolonged, whole‐body demands of 50 km cross‐country skiing (~2 h) to the momentary, explosive actions of ski‐jumping and the running push‐starts in bobsleigh and skeleton. Consequently, the magnitude of active muscle mass and contraction patterns varies markedly. Nordic skiing engages whole‐body concentric and eccentric patterns [4], whereas speed‐ and power‐oriented sports [12]—such as alpine skiing, snowboarding, short‐track speed skating, or skeleton—are characterized by high‐magnitude force production, substantial eccentric loading, and rapid force development from predominantly localized muscle groups [13, 14, 15, 16].

For instance, speed skating combines concentric lower‐limb actions with a deep crouch that restricts blood flow, causing significant deoxygenation asymmetries between straight and cornering phases [17] and leading to local fatigue mismatches. Conversely, alpine skiing involves sustained isometric and eccentric contractions that elevate intramuscular pressure, restricting blood flow and inducing muscle deoxygenation [18], which increases reliance on phosphocreatine stores.

Crucially, these sport‐specific perturbations do not merely scale endurance demands quantitatively but fundamentally alter how aerobic power is utilized. Endurance may act as a direct limiter of sustained power output, a regulator of metabolic demands and recovery kinetics between repeated efforts, or a stabilizing supportive capacity that preserves technical execution under fatigue. Distinguishing between these roles is essential for rational training design and cannot be inferred from exercise duration alone. Success, even in endurance‐limited disciplines (Tiers 1–2), requires highly coordinated neuromuscular and metabolic function [4].

This mechanistic perspective highlights that the prescription of endurance training must be aligned not only with metabolic intensity but also with contraction‐specific fatigue profiles and recovery constraints. Failure to account for these factors risks miscalibrating the true physiological load, leading to suboptimal allocation of training time and recovery resources. Therefore, contraction type is not only a biomechanical variable but also a fundamental determinant of energy system utilization, recovery profiles, and endurance training design.

Combined with the diversity of competition formats, ranging from continuous single‐bout efforts to multiround sprint or technical events, and to cumulative schedules across the Games, these demands necessitate a differentiated classification of training modalities.

- Sport‐Specific: Reproduces biomechanical and metabolic demands with high validity (e.g., cross‐country skiing uphill, speed skating on an ice oval). In Tier 1–2 sports, specific training volume is maximized to drive both central and peripheral adaptation.
- Semispecific: Retains partial biomechanical overlap to target physiological systems while modulating local load (e.g., cycling for speed skaters or inline skating [19]).
- Nonspecific: Biomechanically unrelated activities (e.g., Nordic walking for alpine skiers) that promote systemic adaptations. In Tier 3–5 sports, this is often prioritized to enhance general aerobic power and facilitate recovery without compounding the specific neuromuscular load of technical training [12, 20, 21] a strategy extrapolated from concurrent training research in other domains.

Furthermore, semi‐ and non specific endurance training off‐snow provides a critical means to maintain physical fitness during the competitive season, mitigating the limitations imposed by restricted on‐snow days (~130–150 days/year) and the logistical challenges of international travel [22]. For instance, elite alpine skiers perform 2–4 off‐snow endurance sessions per week during the preparation period and 0–2 during the competition period, prioritizing physiological maintenance while accommodating heavy racing loads. The increased prioritization of such sport‐specific endurance modalities over recent decades represents a fundamental shift in elite training practice.

## 4 The Feeder‐Function Model: Endurance as a Support Mechanism

In Tier 1–2 sports, endurance is a primary limiter, whereas in Tier 3–5 disciplines it typically does not determine competitive outcomes but instead serves as a secondary “feeder function.” In this context, endurance represents an enabling capacity that supports recovery, repeatability, resilience, and long‐term training tolerance within a holistic performance model. This distinction is increasingly critical given the shifting landscape of athlete development; modern athletes often specialize earlier and present with lower accumulated nonsport physical activity compared to previous generations. Consequently, we propose that the “feeder capacity” that was once acquired incidentally must now be explicitly programmed to a greater extent to ensure long‐term training tolerance. Thus, while the aerobic “engine” has remained relatively stable over time, the specificity with which endurance supports recovery, repeatability, and robustness has evolved substantially.

This conceptualization is supported by integrative physiological analyses demonstrating that, in complex winter sports such as alpine skiing, performance emerges from the interaction of multiple systems rather than the dominance of a single physiological factor. While Tier 3–5 events rely predominantly on strength, power, technical skill, and neuromuscular coordination, a well‐developed aerobic system may still confer indirect yet performance‐relevant benefits by enabling higher training density, faster recovery between sessions, and greater tolerance to cumulative load [23, 24].

Empirical support for this is provided by discipline‐specific analyses in freestyle skiing aerials, where competitive success relies on technical precision supported by anaerobic power, explosive strength, and neuromuscular control, while aerobic endurance shows minimal relevance [25]. Furthermore, multivariate analyses in alpine skiing demonstrate that conventional aerobic performance markers, such as VO_2_max, fail to predict competition outcomes [26, 27]. Extending this evidence base, recent champion fitness models integrate weighted indicators of morphology, maximal and explosive strength, anaerobic power, and neuromuscular control [28]. Such frameworks are conceptually consistent with the feeder‐function model, as they explicitly position endurance as a contextual contributor rather than a universal driver, emphasizing that its relative importance must be interpreted within sport‐specific hierarchies. The specific characteristics of these sports are summarized in Table 2.

Furthermore, in Freestyle and Snowboarding, the culture of the sport often necessitates a “natural” endurance stimulus during the snow season; athletes frequently accrue substantial low‐intensity volume through hiking to features and free‐riding. This informal capacity is critical, as it underpins the ability to perform the high volume of technical runs required for skill acquisition. However, with on‐snow time typically limited to ~150 days annually, structured off‐snow endurance training remains essential to bridge the seasonal gap, ensuring a “feeder” capacity is maintained year‐round.

Physiologically, however, a well‐developed aerobic system enhances recovery both between high‐intensity training sessions [29, 30] and during competition. Several mechanisms contribute to this effect. First, moderate endurance training induces an expansion of plasma volume (~6%–16%) and, over longer training periods, increases red‐cell volume (up to 10%) [31, 32], increasing total blood volume and enhancing oxygen‐transport capacity. Second, high‐intensity training typically increases stroke volume [33], further supporting oxygen delivery. Third, repeated bouts of intense intermittent endurance training stimulate capillary growth and endothelial cell proliferation within weeks, with adaptations occurring in both type I and type II fibers [34]. Collectively, these adaptations accelerate recovery from high‐intensity intermittent exercise by improving aerobic response, facilitating clearance of metabolic by‐products [30], enhancing restoration of PCr [30], and strengthening intramuscular buffering capacity [35] to delay the onset of fatigue.

These mechanisms directly improve repeatability, that is, the ability to sustain repeated high‐intensity bouts, which is essential in training drills, qualification rounds, or multiheat competitions. Field‐based observations in alpine skiing show that while neuromuscular fatigue increases across repeated runs, aerobic fitness plays a key role in mitigating this decline by supporting recovery [36]. A robust aerobic base enables larger overall training volumes, supporting technical refinement, tactical preparation, and strength/power development, potentially reducing the risk of overreaching and injury, although discipline‐specific dose–response studies are still needed [37].

From a health perspective, adequate aerobic fitness may support immune competence [38] and attenuate the perception of acute mental fatigue [39], although direct evidence in winter cohorts remains limited. However, excessively high endurance volumes in cold, dry environments are associated with increased prevalence of nonallergic asthma [40], underscoring the need for balanced prescription.

Ultimately, the goal in Tier 3–5 sports is to identify the minimal effective dose of endurance training that maximizes recovery and tolerance without interfering with strength and power development. The central question for coaches is how much, when, and in what form this work should be integrated into in‐season and off‐season programs to maximize transfer without interfering with strength, speed, and technical development. Future research must address these questions through controlled studies in applied athletic environments, combining physiological and performance measures to generate evidence‐based guidelines for integrating and further developing the feeder function model.

## 5 Key Domains of Practice

### 5.1 Individualized Volume–Intensity Architectures

Physiological adaptation to endurance training is fundamentally determined by the interplay between exercise intensity, training volume, recovery, and the athlete's current training status. Exercise intensity refers to the physiological and mechanical demands of exercise relative to an internal or external reference (“anchor”). While external anchors (e.g., speed and power) enable greater standardization, internal anchors (HR, blood lactate concentration, RPE) better reflect biological stress, though they are modulated by hydration, fatigue, and recovery status. Evidence suggests that the predominant anchor may shift with training context—for example, relying on internal anchors during basic endurance training versus external anchors during competition phases [41]. However, the definition of “intensity” remains debated, with ongoing discussions about anchor reliability, model comparability, and their relevance across sports and performance levels [42].

Depending on the sport, federation guidelines, and available resources, exercise intensity is commonly classified using multizone models [43], with standard zones (Z1–Z3) typically based on internal indicators such as heart rate or blood lactate concentration [41, 44, 45, 46, 47, 48, 49, 50, 51, 52, 53, 54]. While distinct physiological thresholds such as Critical Power (CP) and Maximal Lactate Steady State (MLSS) have been proposed to demarcate the heavy and severe intensity domains [55] these testing protocols are logistically difficult to implement in many winter sports environments. Consequently, practical zoning approaches often rely on surrogate markers, assuming that each domain elicits distinct adaptations regardless of the precise anchor used.

In practice, a five‐zone model is widely utilized, partly because many wearable systems and associated software are built around this framework. For scientific comparability, a three‐zone model is often applied, collapsing the intensity spectrum into low (< LT_1_/VT_1_), moderate (LT_1_–LT_2_), and high (> LT_2_) domains [42, 56]. Retrospective analyses of Tier 1–2 sports indicate that low‐intensity training consistently dominates (> 80% of total time), with moderate and high intensities contributing substantially less [56]. While compressing multizone frameworks into three zones facilitates harmonization, this simplification may obscure critical distinctions, for example, the physiological difference between training just below vs. just above LT_2_/CP. Furthermore, the methods used to quantify training intensity distribution (TID) (including heart rate–based time‐in‐zone approaches, session‐goal heart rate, power‐ or velocity‐based measures, and session RPE) often yield divergent results, thereby limiting comparability across studies. For example, in cross‐country skiing, stochastic terrain and frequent intensity transitions may decouple heart rate–derived intensity (i.e., “time in zone”) from external and neuromuscular load, complicating direct comparisons of TID between studies.

An observational study in junior cross‐country skiers [57] reported that ~75% of sessions were performed well below, and ~15%–20% well above, the zone between VT_1_ and VT_2_, with threshold training rarely undertaken. This pattern was termed *polarized*, emphasizing low‐ and high‐intensity work while avoiding the moderate domain. A recent systematic review of elite endurance athletes identified polarized TID in 65 of 175 cases, predominantly in cross‐country skiing and biathlon [56]. In contrast, most analyses in endurance sports report a *pyramidal* TID, with ~75%–80% low intensity, ~15%–20% moderate, and ~5%–10% high intensity, depending on the training phase and analytic method [56]. Thus, relative proportions vary across sports due to both methodological constraints and sport‐specific demands.

Over the past 70 years, endurance training in Tier 1–2 winter sports has primarily focused on accumulating large volumes of low‐intensity exercise as the foundation for athletic preparation. Athletes typically spend the majority of their training time at low intensities, complemented by strategically integrated sessions of moderate‐ and high‐intensity exercise to elicit specific physiological adaptations. Longitudinal evidence from cross‐country skiing indicates that progression from junior to senior world‐class performance is characterized by substantial increases in low‐intensity training volume, whereas gains in VO_2_max are comparatively modest, suggesting that long‐term development at the elite level is driven more by training volume and associated submaximal adaptations than by further increases in maximal aerobic power [58]. This observation supports the notion that long‐term elite development is driven primarily by refined specificity in endurance volume, intensity distribution, and contextual deployment rather than by expansion of aerobic ceilings; accordingly, while low‐intensity training has remained foundational, the past two decades have seen intensified scrutiny of HIIT variants to better understand how different intensity distributions influence performance and their phase‐specific trade‐offs.

The growing research focus on HIIT, combined with the successful application of high‐volume low‐intensity exercise by elite cyclists (including recent Tour de France champions [59]) has likely contributed to renewed scientific and practical interest in prolonged low‐intensity continuous exercise, often termed “Zone 2 training.” In five‐zone models, Zone 2 typically lies at or just below LT_1_/VT_1_ [60], representing a stable, metabolically sustainable range. The primary rationale for extensive Zone 2 exercise is its capacity to stimulate mitochondrial function and enhance fat oxidation [61, 62, 63]. However, the precise boundaries of Zone 2 vary considerably depending on the chosen anchor, and commonly applied markers (e.g., fixed %HRmax, blood lactate thresholds, or surrogate metrics) display substantial inter‐ and intraindividual variability [64]. This variability complicates both training prescription and the interpretation of outcomes.

Nevertheless, in endurance disciplines such as distance running, world‐class performance has been consistently associated with high overall volumes of low‐intensity training [65]. Such sessions allow for increased total training distance, facilitate recovery, and provide a sustained aerobic stimulus that supports cardiovascular, connective tissue, and metabolic adaptations. Accordingly, low‐intensity training volume is widely considered the foundation upon which more specific high‐intensity sessions are layered in successful long‐term training models [65].

Several mechanisms support the inclusion of higher‐intensity training in endurance programs; however, evidence regarding central and peripheral adaptations in elite athletes remains limited and problematic for several reasons: (i) Most data stem from untrained or subelite male populations and/or pretraining assessments, limiting transferability. (ii) In trained individuals, signaling responses such as those involving AMP‐activated protein kinase (AMPK), a key cellular energy sensor, and peroxisome proliferator‐activated receptor gamma coactivator‐1α (PGC‐1α), are often attenuated by prior training status, and the degree of fatigue strongly influences molecular outcomes [10, 66]. (iii) adaptations are modulated by macronutrient availability [67] (iv) comparisons between HIIT and moderate‐intensity continuous training (MICT) are frequently based on work‐matched protocols that may favor HIIT by neglecting the typically large training volumes and distinct adaptations elicited by MICT in elite athletes. (v) The wide range of HIIT formats complicates comparisons across studies. Together, these complexities highlight the challenge of determining how HIIT can be most effectively integrated into a training process dominated by large volumes of low‐intensity exercise.

Both HIIT and MICT promote mitochondrial biogenesis and enhance oxidative enzyme activity via overlapping signaling pathways, including AMPK, p38 MAPK, and PGC‐1α [68]. Consequently, both modalities improve oxygen utilization, contributing to improvements in VO_2_max. Depending on the protocol, HIIT induces greater acute metabolic perturbations within shorter durations [69] and recruits a broader motor‐unit pool (including both type I and type II fibers), thereby promoting mitochondrial adaptations in fibers less challenged during MICT [68]. By contrast, MICT predominantly activates type I fibers under a more stable metabolic environment [68]. In addition, HIIT appears more effective in augmenting stroke volume [33], a key determinant of oxygen transport and an important central adaptation supporting endurance performance.

These complementary mechanisms explain why different training intensity distributions (TIDs), with varying proportions of low‐, moderate‐, and high‐intensity exercise, can stimulate both central and peripheral adaptations. Sport‐specific demands shape these distributions: for example, a pyramidal TID is common during preparation phases, whereas a more polarized profile is often observed closer to competition [56]. Current evidence does not conclusively favor one pattern, as methodological heterogeneity and the retrospective, largely descriptive nature of available studies limit generalizability [56]. Therefore, effective preparation requires a flexible, periodized balance of intensities that aligns with sport‐specific demands, athlete responses, and the timing within the competitive season.

Looking forward, individualized volume‐intensity architectures are likely to move beyond population‐based templates toward athlete‐ and sport‐specific frameworks prescribing principles. For example, in cross‐country skiing, individualized prescriptions may distinguish athletes with high fractional utilization of low durability from those with greater fatigue resistance but lower maximal aerobic power, leading to different emphases on low‐intensity volume versus high‐intensity training. In contrast, in sports such as biathlon or Nordic Combined, intensity architectures may need to account for the interaction between endurance load and precision‐ or technique‐dependent tasks (e.g., shooting accuracy or ski jumping performance), favoring intensity distributions that minimize residual fatigue while preserving technical consistency. Similarly, in long‐ and short‐track speed skating, the training architecture is defined not only by intensity but by the critical ratio of specific on‐ice work to general off‐ice conditioning (predominantly cycling). Due to the high intramuscular forces and restricted blood flow inherent to the skating crouch, elite skaters cannot achieve total aerobic volumes comparable to cross‐country skiers solely through specific training; thus, the optimal architecture must carefully balance aerobic/cardiovascular loading via non‐specific modes (e.g., cycling) with the maintenance of technical integrity on the ice.

In Tier 3–5 sports, individualized integration of endurance training may be guided less by maximal aerobic markers and more by indicators of recovery kinetics, neuromuscular function, and tolerance to cumulative load. For instance, alpine skiers with high eccentric strength demands may benefit from endurance prescriptions that prioritize low‐impact modalities and internal‐load regulation, whereas freestyle skiers and snowboarders competing in heat‐based multiround formats may require individualized microcycles emphasizing repeatability and rapid recovery between heats.

Collectively, these examples highlight that individualized training in winter sports is unlikely to converge on a single optimal model; instead, future practice will require flexible frameworks that integrate sport‐specific demands, athlete typologies, and real‐time monitoring to dynamically adjust volume–intensity relationships across the season.

Finally, the temporal distribution of intensity (specifically during the taper) is a critical component of the training architecture [70]. While training errors far from competition can often be mitigated, suboptimal preparation during the final tapering phase is particularly detrimental. A recent meta‐analysis of taper strategies in endurance athletes confirmed consistent benefits but emphasized that poorly aligned or improperly executed tapers can negate prior training gains and blunt competition outcomes [70]. Current best‐practice recommendations generally involve a 40%–60% reduction in training volume over ~7–14 days while maintaining training intensity to preserve neuromuscular and cardiovascular adaptations [71, 72]. However, for winter endurance athletes, this phase is uniquely complicated by the necessity of simultaneous altitude acclimatization and travel recovery. For instance, guidelines for the Beijing 2022 Games recommended synchronizing a 7–10 day taper with acclimatization at competition altitude (~1700 m), explicitly prescribing high‐intensity bouts to counteract the reduced speeds often associated with initial altitude exposure [73].

Furthermore, variable weather conditions in winter sports frequently lead to competition delays or rescheduling, challenging the timing and precision of the taper. These uncertainties demand agile taper models that can be dynamically adjusted to environmental conditions and competition delays, ensuring physiological readiness while avoiding premature peaking or detraining.

Future research should prioritize developing validated and reliable methods to prospectively prescribe training volume, refine the characterization of mid‐range intensities, and elucidate how different volume–intensity architectures interact with sport‐specific demands, athlete tiers, and long‐term adaptation.

### 5.2 Beyond Traditional Metrics: Operationalizing Emerging Performance Constructs

While classical endurance training models have prioritized metrics such as V̇O_2_max, lactate threshold, and movement economy [74, 75, 76, 77], emerging competitive demands including prolonged race durations, multiday events, and environmental stressors highlight the need to consider supplementary dimensions of performance. Current discussions center on contextualizing and operationalizing a fourth performance dimension in endurance sports, beyond traditional metrics [78]. This dimension—variously termed as durability [79], fatigability [80, 81], (physiological) resilience [82], and (high‐intensity) repeatability [79] offer a more nuanced (sport‐specific) understanding of how athletes tolerate cumulative load, recover between efforts, and sustain function under physical, psychological, or environmental stress. Proposed operationalizations include lab‐based fatigability protocols (e.g., gross efficiency decline postfatigue), field tests for repeatability (e.g., repeated time trials), and resilience indices tracking recovery kinetics, with ongoing debates on sport‐specific thresholds and integration into periodized training [78].

Notably, disciplines such as cross‐country skiing and biathlon combine prolonged duration with an inherently intermittent intensity profile driven by undulating terrain, frequent changes in speed and gradient, and transitions between subtechniques. This “variable load” nature requires athletes to repeatedly generate high power outputs on climbs, recover rapidly on descents, and maintain technical efficiency under fluctuating intensities. Such conditions demand a complex integration of durability, high‐intensity repeatability, and physiological resilience, highlighting that performance in these sports is not solely determined by interval‐based capacity, but also by the ability to sustain power output under terrain‐driven stochastic workloads.

Recent evidence in well‐trained runners indicates that training characteristics such as higher overall volumes, regular long runs, and strength training are associated with superior durability of running economy and attenuated neuromuscular performance decrements during prolonged exercise [83]. These traits are increasingly relevant for tailoring periodization, individualizing recovery strategies, and optimizing performance in real‐world scenarios, including prolonged races (e.g., 50 km cross‐country skiing event or the 20 km Individual in biathlon) and formats requiring multiple rounds or stages (e.g., cross‐country skiing and ski mountaineering sprint races, short‐track speed skating events, and ski cross), where athletes must manage cumulative fatigue while repeatedly producing high‐level efforts under varying tactical and environmental constraints.

Overall, these emerging constructs remain operationally underdefined, with limited consensus on assessment protocols and context‐specific metrics [64]. Moreover, the intermittent characteristics of certain winter sports further complicate the definition and testing of these traits under controlled conditions. This conceptual and methodological ambiguity raises a practical challenge: without standardized definitions and validated assessments, it remains unclear which underlying qualities (e.g., metabolic, neuromuscular, or cognitive) should be prioritized, and how training interventions can be optimally designed to develop these specific capacities.

Addressing this gap is therefore a key priority for future winter sports research and applied sports science. Future work must establish standardized, sport‐specific methods to quantify each construct and disentangle their physiological and psychological determinants. Importantly, laboratory‐based data alone are insufficient to capture the complexity of endurance performance, particularly under stochastic, prolonged, and ecologically valid conditions. Accordingly, field data from validated wearable technologies, interpreted within sport‐specific contexts, are essential for understanding how these constructs manifest across endurance disciplines.

### 5.3 Strategic Figure Use of Multimodal Stress Stacking

The strategic combination of physiological and environmental stressors (“stress stacking”) is increasingly utilized in elite endurance training to amplify adaptations beyond single‐stimulus interventions. The rationale involves delivering multiple synchronized and complementary stimuli to the body's adaptive systems, targeting distinct steps in the oxygen transport chain, metabolic pathways, and neuromuscular function in a coordinated manner. Stacking can be applied progressively (linear), simultaneously (concurrent), chronically, or concentrated within short, demanding blocks (shock microcycles, that is, brief periods of deliberately intensified training load designed to exceed normal recovery capacity). Such blocks accumulate high‐load stimuli over consecutive days [84, 85], inducing acute fatigue followed by a supercompensatory response. The specific format of stacking depends on the targeted physiological systems, the competition calendar, and athlete readiness.

Hypoxic stimuli, delivered via natural altitude or artificial induction, remain central, with the classical live‐high/train‐low (LHTL) model aiming to stimulate erythropoiesis while preserving training intensity [86]. Modern applications include intermittent hypoxic exposure (IHE) to promote hematological adaptations [87]; repeat‐sprint training in hypoxia (RSH) to stress glycolytic pathways and enhance buffering capacity [88]; and combined hypoxia‐heat protocols intended to couple hematological benefits with plasma‐volume expansion and peripheral adaptations [89]. Because hypoxia modifies the relationship between external workload and internal physiological strain, individualized indicators of internal load (e.g., cardiovascular responses) provide a more valid quantification of the effective training dose than generic external metrics during altitude training, and show stronger associations with physiological adaptation [90].

Methods involving the direct manipulation of inspired air, such as breathing elevated fractions of oxygen (hyperoxia) during training or carbon monoxide inhalation to induce transient hypoxemia, have also been explored. However, their use remains actively debated, and substantial ethical and safety concerns argue against supporting these approaches as ergogenic aids in elite sport [91, 92].

Heat training, initially developed for acclimatization to hot climates, is now studied for its ergogenic potential under temperate conditions. Its primary mechanism appears to be plasma volume expansion, which improves stroke volume, cardiovascular stability, and thermoregulation [93]. A prominent form of “multiple stress stacking” involves combining altitude and heat exposure. However, current evidence does not conclusively demonstrate superior performance benefits at sea level compared to conventional single‐stressor interventions [89]. Key challenges remain in defining optimal dosing regimens for both exercise and environmental exposures, and the potential advantages of sequential versus concurrent heat and altitude training require clarification [89]. Moreover, the relevance of heat–hypoxia interactions for winter‐sport athletes competing in cold environments remains unknown.

Blood flow restriction (BFR) training may provide a novel stimulus for endurance athletes by inducing local hypoxia and metabolic stress at low intensities, potentially promoting both hypertrophic and oxidative adaptations [94, 95]. Although well established in rehabilitation and resistance training, its application in elite endurance sport remains limited. Early trials in trained runners and rowers suggest BFR may enhance VO_2_max and neuromuscular performance [96, 97]. While heat and hypoxia exposures are established strategies for preacclimatization, more controversial methods such as repeated carbon monoxide (CO) inhalation have also been discussed for stimulating hematological adaptations [98], though they carry substantial uncertainty and risk [92]. Consequently, the application of any such environmental stressor (especially CO) requires strict medical governance and ethical oversight to prevent uncontrolled practices and ensure athlete safety [92].

### 5.4 Concurrent Training: Integrating Strength and Power Elements

The role of strength and power training differs fundamentally across the winter sport tiers. In Tier 1–2 (Endurance‐Limited) sports, resistance training is typically implemented as a complementary stimulus to enhance neuromuscular efficiency, preserve technique under fatigue, and augment sprint and power characteristics [99, 100]. In contrast, in Tier 3–5 (Feeder‐Function) disciplines such as alpine and freestyle skiing, snowboarding, and ice hockey, muscular strength and power constitute primary performance determinants rather than supportive capacities. In these sports, such qualities drive the capacity to generate propulsive impulses, tolerate repeated eccentric loading, and maintain robustness under high mechanical stress, whereas endurance training primarily supports recovery between bouts and sustains repeated high‐intensity neuromuscular efforts across training sessions and competition.

For endurance‐limited athletes (e.g., cross‐country skiers, biathletes), the rationale for integrating resistance training is to improve the neuromuscular component of efficiency and performance robustness. Decisive race moments (e.g., mass‐start surges and sprint finishes) require high short‐duration force and power production [101, 102]. Moreover, greater maximal strength can reduce the relative force requirement of submaximal movement cycles, potentially improving movement economy and delaying technique deterioration as fatigue accumulates [99, 100]. This is particularly relevant in the classical subtechnique of double poling, where propulsive phases at high speeds are extremely brief (≤ 0.20 s), emphasizing rapid force development rather than force expressed at low contraction velocities [102, 103]. Accordingly, strength interventions in this tier often aim to prioritize neural and musculotendinous adaptations (e.g., improved motor‐unit recruitment and rate coding, and increased musculotendinous stiffness) with minimal hypertrophy to preserve power‐to‐mass characteristics [100, 104, 105].

In Tier 3–5 disciplines, the training objective is more directly oriented toward maximizing force production and mechanical load tolerance. In gravity‐dependent snow sports (e.g., alpine skiing, and in many of the events in freestyle skiing and snowboarding), athletes are exposed to high ground‐reaction forces, sustained eccentric loading, and rapid force fluctuations while navigating irregular terrain and challenging course features [106]. In this context, training of strength and power contributes not only to performance‐relevant actions (e.g., start acceleration, forceful turn initiation, and impulse generation), but also to robustness and injury‐risk mitigation by improving the capacity to absorb and attenuate repeated high‐load events [107]. This is especially critical for landing‐ and impact‐dominant tasks in freestyle and snowboarding disciplines and for repetitive high‐frequency loading in alpine events [108]. Similarly, in intermittent ice sports (e.g., ice hockey and figure skating), explosive power and reactive strength are central for repeated accelerations, decelerations, jumps, and change‐of‐direction actions [109, 110, 111, 112]. Here, the “Feeder Function” of endurance is important primarily insofar as it supports recovery and maintains output across repeated high‐intensity bouts, congested competition schedules, as well as supporting “the ability to train,” rather than serving as the primary limiter of maximal performance.

Across tiers, the transfer of strength and power adaptations to sport performance is modality‐ and context‐dependent. Current evidence suggests that heavy resistance training, explosive/ballistic training, and plyometric modalities can improve strength‐power qualities and, in endurance‐dominant athletes, may translate to improved economy or performance in tasks that are limited by short force‐production windows [104, 107, 113]. In contrast, isolated low‐load or non‐specific interventions (e.g., static core training) often demonstrate limited transfer to dynamic sport performance, underscoring the importance of specificity and sufficient loading [107].

Effective implementation also requires managing cumulative fatigue and the potential “interference” between endurance and strength/power stimuli, particularly when large endurance volumes are combined with frequent strength sessions [114, 115]. In Tier 1–2 sports, the practical concern is commonly to retain strength/power development without compromising key endurance training quality. In contrast, for Tier 3–5 athletes, excessive endurance volume and poorly timed aerobic work may compromise neuromuscular quality by increasing fatigue and shifting adaptation emphasis away from high‐velocity force production. Accordingly, periodization strategies should reflect tier‐specific priorities: Tier 1–2 athletes may integrate strength to support efficiency and durability while maintaining high aerobic volume, whereas Tier 3–5 athletes may employ targeted aerobic “microdoses” to support recovery and work capacity without diminishing explosive adaptations. Future research should clarify dose–response relationships across tiers, including how in‐season strength maintenance interacts with technical demands, mechanical loading, and fatigue resilience under ecologically valid competitive conditions.

### 5.5 Athlete‐Centered Factors Within a Holistic Training Model

Optimizing endurance training for elite athletes requires integrating sport‐specific demands with a nuanced understanding of athlete‐centered factors. These determinants, which vary in their degree of trainability, shape both the ceiling of performance potential and the pathways through which adaptation can be realized.

Trainable biological factors represent physiological systems and tissues that can be modified through targeted interventions, although their adaptability remains bounded by the nontrainable framework described in Figure 1. These include cardiovascular and muscular adaptations, as detailed in a companion paper of this topical issue (see Paper 1). Beyond these central systems, pulmonary function is partially adaptable; while structural lung volumes are largely constrained in adults, endurance training improves ventilatory efficiency and can modestly enhance gas‐exchange capacity, supporting oxygenation under high metabolic demand [116]. Simultaneously, vascular adaptations (e.g., increased skeletal‐muscle capillary density and improved endothelial function) facilitate oxygen delivery, distribution, and peripheral extraction [116]. Thermoregulatory capacity demonstrates significant plasticity, particularly through heat acclimation. Key adaptations include plasma volume expansion and refined sweating responses, alongside enhanced cardiovascular stability, which collectively improve tolerance to thermal stress and help preserve performance under demanding conditions [117, 118]. In parallel, gastrointestinal function is also trainable: repeated exposure to carbohydrate intake during exercise can improve gastric emptying, intestinal absorption, and tolerance, thereby reducing GI distress and supporting hydration and fueling during prolonged winter endurance events [119, 120]. Finally, in winter endurance sports such as cross‐country skiing, neuromuscular adaptations optimize inter‐ and intramuscular coordination, refining technical efficiency and force distribution in complex multijoint tasks [4, 121].

Complementing these physiological systems, the “24‐h athlete” concept [122] integrates psycho‐behavioral, environmental, and nutritional dimensions into the performance model. This perspective emphasizes that adaptation and performance are shaped not only by training load, but also by lifestyle behaviors and mental competencies expressed across the full day. Psychological skills, including effort regulation, attentional control, and decision‐making under fatigue, are increasingly recognized as trainable capacities that can enhance resilience and day‐to‐day performance. Mindfulness‐based interventions have gained attention for enhancing athlete well‐being [122, 123, 124]. Concurrently, sleep is a foundational recovery behavior: chronic sleep restriction impairs recovery and adaptation, degrades cognitive performance, and elevates injury risk [125, 126].

Nutrition further underpins recovery, immune function, and bone health. Maintaining energy availability mitigates the risk of Relative Energy Deficiency in Sport (REDs) [127, 128], while adequate intake of carbohydrates [129] and protein [130] supports fueling, repair, and adaptation (see Paper 5). Furthermore, cold and altitude exposure increase energy expenditure, requiring precise hydration and micronutrient management, with particular attention to vitamin D sufficiency in winter athletes [131].

The effectiveness of any training model is also shaped by the athlete's support ecosystem, spanning personal resources (e.g., self‐management/−regulation, motivation), private support (e.g., family, personal coaches, medical care), and institutional infrastructure (e.g., federation facilities, multidisciplinary teams, funding). Although support organization is partly discipline‐specific, the extent of individualization is dictated by performance status and resource accessibility. Consequently, two athletes within the same discipline may operate under markedly different models depending on their support systems.

Taken together, these athlete‐centered factors underscore the multidimensional and interdependent nature of endurance performance. While nontrainable determinants establish the biological ceiling, trainable physiological systems provide the functional substrate; ultimately, psycho‐behavioral, nutritional, environmental, and support factors determine how effectively this potential is realized and sustained. Future research should adopt integrative approaches to examine how these domains interact dynamically across an athlete's career, including how lifestyle, support structures, and psychological competencies influence long‐term adaptation, resilience, and health.

### 5.6 Technology and Mechanistic Frontiers: From Monitoring to Modeling

Technological and analytical advances are fundamental to operationalizing the athlete‐centered concepts outlined in Sections 2.3 and 2.4, shifting the paradigm from passive monitoring to active performance modeling. Multisensor platforms enable the quantification of power output, movement patterns, and environmental conditions, while advanced GNSS and biosensor technologies can track velocity, hydration status, and, in specific contexts, muscle oxygenation, core temperature, and gastrointestinal function [132, 133, 134]. Crucially, the utility of these tools depends on their validation across diverse environmental conditions (e.g., temperature extremes, moisture). These measures allow key constructs, such as training intensity distribution, durability, repeatability, and recovery capacity, to be assessed under ecologically valid training and competition conditions, enabling real‐time adjustments and precise long‐term load management.

Artificial intelligence and machine learning are increasingly applied to transform large datasets into predictive insights [135, 136], while the emerging concept of “digital twins” holds the potential to integrate with physiological, biomechanical, equipment (e.g., aerodynamics, gliding mechanics, ski/boot/binding stiffness and base structure), course/venue geometry, and environmental data to simulate adaptive scenarios [137, 138]. Mechanistic approaches such as multiomics, polygenic profiling, MRI, and microbiome analysis [139] further deepen the understanding of how diverse subsystems interact during training and recovery.

Despite this potential, translation into daily practice remains constrained by cost, standardization, technical limitations in field settings, and ethical considerations. Accordingly, current AI applications should be framed as decision‐support tools that advance, rather than replace, coaching expertise and athlete self‐regulation. From Cortina 1956 to Milano‐Cortina 2026, technology has evolved from simple observation to a central component of individualized performance modeling, provided it is applied transparently, responsibly, and in alignment with athlete‐centered principles.

### 5.7 Main Factors of Competitive Success and the Evolving Athlete Typology

Across Olympic winter sports, competitive success is shaped by a constellation of interrelated determinants. While sport‐specific demands vary, several core factors consistently emerge as decisive. These range from foundational physiological determinants (e.g., V̇O_2_max, lactate threshold, power/speed capacities, and movement economy) and strategic elements (e.g., pacing proficiency and substrate utilization efficiency) to crucial psycho‐physiological qualities (e.g., resilience, durability, fatigability, and repeatability). Together, these form the framework within which tactical execution, technical precision, and mental control are expressed.

Historically, athlete profiling was anchored primarily in laboratory‐derived metrics (e.g., maximal aerobic power and threshold indices), and training models were discipline‐specific and tradition‐driven. Today, advances in wearable technology, real‐time analytics, and sport‐specific field testing have expanded both the scope and resolution of performance profiling. This evolution has highlighted the importance of “extended” qualities such as durability under cumulative fatigue, repeatability of high‐intensity efforts, and resilience to environmental and psychological stressors. These allow more precise identification of limiting factors across both endurance‐ and nonendurance‐dominant sports. Accordingly, the major paradigm shift over recent decades lies in the increased specificity, monitoring, and application of multistacking training methods, rather than in further enhancement of maximal aerobic power.

This broadening evidence base has also refined athlete typologies. Rather than classification solely by event or discipline, athletes are increasingly segmented by physiological profile, limiting factors, and adaptive tendencies. For example, endurance specialists with high fatigue resistance but limited sprint repeatability differ markedly from power‐oriented athletes who use endurance training primarily to enhance recovery, training tolerance, and mental resilience. Such typological distinctions directly inform training prescription and periodization.

In essence, competitive success toward 2026 and beyond will rely not only on maximizing established determinants but also on contextualizing them within sport‐specific demands, the athlete's typological profile, and a demand‐driven, athlete‐centered, data‐supported training process. The convergence of refined performance science, individualized profiling, and the strategic application of endurance conditioning, whether as a primary limiter or as a feeder capacity, marks a decisive departure from previous paradigms.

**FIGURE 3:**
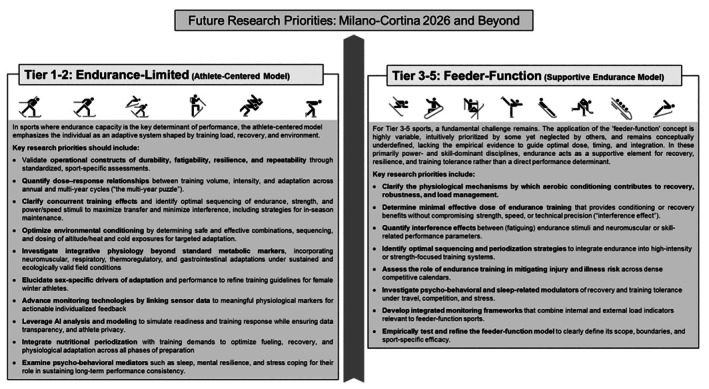
Summary of future research priorities for Olympic winter sports in preparation for Milano Cortina 2026 and beyond. The framework distinguishes Tier 1–2 (Endurance‐Limited) disciplines (*athlete‐centered model*), where priorities include validating operational constructs (e.g., durability, fatigability, resilience, repeatability), quantifying multiyear dose–response relationships, optimizing concurrent and environmental conditioning strategies, and advancing interpretable monitoring/AI‐enabled decision support. In Tier 3–5 (Feeder‐Function) disciplines (*supportive endurance model*), priorities focus on defining the mechanistic role and minimal effective dose of endurance training to support recovery and training‐load tolerance without compromising neuromuscular or technical qualities, quantifying interference effects, and refining integration strategies across dense competitive calendars.

Nevertheless, based on the synthesis of the current literature, several research avenues are summarized in Figure 3 and appear particularly relevant for advancing understanding and practice as we look toward 2026 and beyond.

## 6 Conclusion

The journey from Cortina 1956 to Milano‐Cortina 2026 is not merely a story of scientific progress, but of shifting paradigms. Preparation has moved from a generic reliance on volume accumulation to a more nuanced understanding of training architecture, where endurance serves dual roles: as the primary metabolic engine in traditional aerobic sports and as a critical “feeder” capacity supporting high‐neuromuscular loads in technical disciplines.

Future performance leaps are unlikely to stem from a single new metric or technology; rather, they will emerge from integration—seamlessly linking physiology with psycho‐behavioral factors, laboratory insights with field practice, and data streams with the “24‐h athlete” h. While emerging tools now provide the resolution to measure durability, resilience, and recovery in real‐time, they must remain servants to the coaching process, not masters of it. Ultimately, the goal remains unchanged: to build robust, resilient, and repeatable athletes—but today, this is an endeavor informed by data, centered on the individual, and defined by holistic integration.

## 7 Perspectives

From Cortina 1956 to Milano‐Cortina 2026, the landscape of endurance preparation in Olympic winter sports has transformed from an empirically guided art to a more scientifically grounded, athlete‐centered discipline. Yet, as our understanding deepens, the primary challenge becomes integrating biological, psycho‐behavioral, environmental, nutritional, and technological domains into a cohesive performance model. Future research, as summarized in Figure 3, should be tailored to address specific sport demands.

For Endurance‐Limited disciplines (Tier 1–2), priority lies in operationalizing “robustness”—validating constructs of durability and repeatability that transcend traditional physiological capacity. Conversely, for Feeder‐Function sports (Tier 3–5), the field needs to define the minimal effective dose of endurance required to support recovery and training tolerance without compromising maximal strength and neuromuscular explosiveness. While these paradigms are forged in the winter context, their utility extends well beyond the Games; the “Feeder‐Function” framework offers high translational value for summer disciplines characterized by similar physiological conflicts (e.g., rowing, kayaking, track cycling), suggesting that insights refined for Milano‐Cortina 2026 may ultimately reshape preparation across the wider Olympic spectrum. Ultimately, the next decade of sports science should move beyond isolated metrics toward holistic, advanced analytical models (e.g., AI‐supported) that link sensor data with meaningful physiological markers. By clarifying these context‐dependent mechanisms, research will empower practitioners to optimize the intricate balance between training load and recovery, ensuring athletes arrive at the Games not just fit, but resilient and better primed for peak performance.

## Funding

The authors have nothing to report.

## Conflicts of Interest

The authors declare no conflicts of interest.

## Data Availability

The data that support the findings of this study are available from the corresponding author upon reasonable request.
